# Vascular parameters and endothelin-1 measurements in glaucoma patients with low- and high-tension optic disc hemorrhages

**DOI:** 10.1038/s41598-023-31682-w

**Published:** 2023-03-28

**Authors:** Izabela N. F. Almeida, Elise Taniguchi, Cecília Victoria Agapito Tito, Diego Torres Dias, Michele Ushida, Syril Dorairaj, Robert Ritch, Sérgio H. Teixeira, Augusto Paranhos, Carolina P. B. Gracitelli, Cristiane Kayser, Tiago Santos Prata

**Affiliations:** 1 Department of Ophthalmology, Federal University of São Paulo, Rua Dr Jose Rodrigues Alves Sobrinho, 125, Alto de Pinheiros, São Paulo, SP 05466-040 Brazil; 2 Rheumatology Division, Federal University of Sao Paulo, Sao Paulo, SP Brazil; 3 Glaucoma Unit, Hospital Medicina dos Olhos, Osasco, SP Brazil; 4 Department of Ophthalmology, Mayo Clinic, Jacksonville, FL USA; 5 Einhorn Clinical Research Center, New York Eye and Ear Infirmary of Mount Sinai, New York, USA; 6 Centro de Estudos Alcides Hirai, Ver Mais Oftalmologia, Vinhedo, São Paulo, Brazil

**Keywords:** Eye diseases, Optic nerve diseases

## Abstract

This prospective study aimed to compare vascular parameters (endothelin-1 [ET-1] blood levels, laser Doppler imaging [LDI] of distal phalanxes, and nailfold capillaroscopy) between open-angle glaucoma patients with low- and high-tension optic disc hemorrhages (LTDH and HTDH, respectively). The 33 enrolled patients (mean age, 62.3 ± 13 years) were classified as LTDH or HTDH if they presented at the time of DH detection an intraocular pressure (IOP) < 16 mmHg or ≥ 16 mmHg, respectively. Demographic and ophthalmological data, ET-1 concentrations, LDI (before and 1, 10, and 20 min after cold stimulation), and nailfold capillaroscopy findings were evaluated. The ET-1 blood level was 65% higher in the LTDH (2.27 ± 1.46 pg/ml) than in the HTDH (1.37 ± 0.57 pg/ml; p = 0.03) group. Moreover, there was a statistically significant negative correlation between ET-1 blood concentration and IOP at the time of DH detection (r = −0.45, p = 0.02). Blood flow measurements 10 and 20 min after cold stimulation were lower in the LTDH group than in the HTDH group (p < 0.01). Patients developing DH with lower IOPs have higher ET-1 blood levels and more peripheral vascular dysfunction as estimated by LDI than those with higher IOPs. These findings suggest that distinct underlying mechanisms may be involved in patients developing DH within different IOP ranges.

## Introduction

Glaucoma is the leading cause of irreversible blindness in the world^1^. Despite a growing number of studies, the etiology of open-angle glaucoma (OAG) remains unclear^2^. Although elevated intraocular pressure (IOP) remains the most important risk factor for glaucoma^3^, many patients may develop glaucoma or experience disease progression despite IOP values within the normal range^4–7^. As OAG is a multifactorial disease, various studies have associated vascular factors with OAG pathogenesis^8–10^.

Optic disc hemorrhage (DH) has been consistently associated with glaucoma^11–13^. DH is usually detected in patients with normal-tension glaucoma but can also be observed in patients with primary open-angle glaucoma^14^. Although not fully elucidated, two main mechanisms have been proposed to explain DH etiology. In brief, the mechanical theory involves pressure-induced damage ultimately leading to the rupture of small peripapillary blood vessels. The vascular theory involves blood-retinal barrier breakdown at the border of the optic disc due to abnormal levels of endothelin-1 (ET-1) and matrix metalloproteinases^15–18^.

Several qualitative and quantitative vascular parameters have been investigated in glaucoma patients, such as elevations in ET-1 levels^19–21^ and nailfold capillaroscopy (NC) abnormalities^22–24^. Another potential alternative for vascular analysis includes laser Doppler imaging (LDI), a noninvasive technique that measures superficial cutaneous microvascular blood flow^25^. Although often used to access blood perfusion in systemic sclerosis studies^26,27^, to the best of our knowledge, until now, there have been no studies using LDI in glaucoma.

As DHs may present in a wide range of IOP values, we divided our study population into two groups: patients presenting DHs with treated IOPs in the low teens (low-tension disc hemorrhage [LTDH]) and patients presenting DHs with higher IOP values (high-tension disc hemorrhage [HTDH]. The rationale for such an approach relies on a recent study from our group, in which we observed that patients with LTDH and HTDH differed significantly regarding epidemiological, systemic, and ocular characteristics^28^. Among these differences, symptoms of vascular dysregulation were more common in patients with LTDH. In the present study, we hypothesized that vascular parameters may also differ significantly between these two distinct groups. Therefore, we sought to compare vascular function-related parameters, as assessed by ET-1 blood levels, LDI of distal phalanxes, and NC, between treated OAG patients with LTDH or HTDH.

## Material and methods

In this prospective cross-sectional study, participants were recruited from the Federal University of São Paulo (São Paulo, Brazil) and Hospital Medicina dos Olhos (HMO, Osasco, Brazil). All protocols were approved by the Institutional Review Board of the Federal University of Sao Paulo (CEP 1.794.726) and were performed in accordance with the ethical standards of the Declaration of Helsinki. Additionally, written informed consent was obtained from all participants.

### Participants

We examined consecutive OAG patients for the presence of DH. The inclusion criteria were (1) clinical diagnosis of glaucoma, defined by abnormal optic disc excavation with corresponding visual field loss, (2) best corrected visual acuity equal to or better than 20/60, and (3) open angles on gonioscopy. The exclusion criteria were (1) other major retinal pathologies, (2) refractive error exceeding ± 6.00 diopters sphere or ± 3.00 diopters astigmatism, (3) optic disc torsion more than 15° or a tilt ratio > 1.3 (maximum-to-minimum optic disc diameter), (4) diagnosis of a vascular or connective tissue disease, and (5) use of any vasoactive medication.

All participants completed a basic medical questionnaire, and their clinical characteristics (best corrected visual acuity, IOP, gonioscopy, lens status, and optic disc appearance) were assessed during the first appointment. Tonometry was performed using a Goldmann applanation tonometry, gonioscopy was performed using a four-mirror goniolens (Volk Optical, Mentor, OH, USA), and dilated fundus examination was performed using a noncontact 90D fundus lens (Ocular, Bellevue, WA, USA). In addition, the medical records, including demographic characteristics (age, sex, and ethnicity), preexisting medical conditions (diabetes mellitus, systemic hypertension, vascular disorders, connective tissue disorders), past ocular surgeries, and ocular and systemic medications of the participants were assessed.

Participants who met the inclusion criteria and did not meet any of the exclusion criteria underwent specific tests in the six-month study period.

Patients were then divided into LTDH and HTDH groups according to the median IOP of the study population (16 mmHg). Details of this methodology have already been published elsewhere^28^. Patients presenting with an IOP < 16 mmHg at the time of DH detection were classified as LTDH, and those with an IOP ≥ 16 mmHg were classified as HTDH. This methodology (median split) has been previously adopted in the Early Manifest Glaucoma Trial, where patients were divided into two groups, low-tension and high-tension IOP, for assessment of glaucoma progression predictors^29^. Finally, clinical and ocular data at the time of DH detection were compared between patients with LTDH and those with HTDH. Whenever both eyes were eligible, only one was randomly selected for analysis.

### Endothelin-1 measurements

Blood samples were obtained from the antecubital vein, preferably in the morning and after 5 to 10 min of rest in a sitting position. After 30 min at room temperature, serum was separated by 15 min of centrifugation, immediately aliquoted in 0.3-ml cryovials, and properly stored until further processing^30^. The concentration of ET-1 was measured using an enzyme-linked immunosorbent assay (R&D, Inc., Minneapolis, MN, USA) according to the manufacturer’s instructions.

### Nailfold capillaroscopy

NC was performed by the same rheumatology specialist in a temperature-controlled (24 °C) laboratory after an acclimatization period (1 h) using a widefield stereomicroscope (SZ40, Olympus) under 10–25× magnification. All fingers were evaluated, except for the thumbs. The following parameters were analyzed: the presence of hemorrhages and ectasia.

### Laser Doppler imaging

In the same temperature-controlled laboratory, LDI was performed. The blood flow from four fingertips (excluding the thumb) of the non-dominant hand was measured using LDI (Moor LDI-VR, Moor Instruments, Axminster, UK) before and after cold stimulation by immersion of both hands in water at 15 °C for 60 s, followed by monitoring of the blood flow at 1, 10, and 20 min after the cold stimulus.

The device uses a red helium–neon laser operating at 633 nm with a skin penetration of approximately 1 mm in depth. The scan speed was 4 ms/pixel, and the scanning time was 3 min and 15 s for each measurement. The mean fingertip blood flow (FBF) of the four fingertips was obtained using an automated software system (Moor LDI system software V5.2), and the average was calculated to determine the global mean. Blood flow was recorded in arbitrary perfusion units (PU)^26^. Delta FBF (Δ-FBF) was also calculated, which represents the percentage difference between post- and pre-cold stimulus FBF^31^.

### Statistical analysis

Descriptive analysis was used to present demographic and clinical data. Descriptive statistics included mean and standard deviation values. Data with normal distribution were analyzed using the independent samples t-test and those without normal distribution using the Mann–Whitney test. Categorical data were analyzed using the chi-square test.

For the comparison of ET-1 levels between patients with LTDH and HTDH, we used the Welch test^32^ to account for unequal variances in these two independent groups. The correlations of ET-1 levels with IOP values and LDI results (at 10 and 20 min) were also investigated (Pearson correlation). Scatter plots were constructed including LOESS (Local Regression Smoothing) trendlines (a span of 80% was adopted) to smooth out fluctuations in data and show a pattern or trend more clearly. Span represents the proportion (expressed as a percentage) of the total number of points that contribute to each local fitted value.

For this study, we chose the correlation between ET-1 levels and IOP values as our main outcome for sample size calculation. Considering an alpha value of 0.05 and an estimated correlation coefficient of 0.50 between these two parameters, a minimum of 29 participants was required to achieve a sample power of 80%. Statistical significance was set at p < 0.05. Computerized analyses were performed using MedCalc software (MedCalc, Inc., Mariakerke, Belgium).

## Results

A total of 33 patients with a mean age of 62.3 ± 13 years and a mean IOP of 14 ± 4 mmHg were included. The mean IOP values were 12.0 ± 2 and 18.6 ± 3 mmHg in the LTDH and HTDH groups, respectively (p < 0.01). No significant differences were found regarding age, gender, race, central corneal thickness, and visual field (VF) mean deviation index between the two study groups (p > 0.05 for all comparisons). Table 1 summarizes the demographic and clinical findings of both groups.

**Table 1:** Demographic and ocular characteristics of study participants.

Variables*	LTDH (n = 22)	HTDH (n = 11)	p-value
Age (years)	60.6 ± 12.8	65.5 ± 12.4	0.30
Gender (F/M)	86.4%/13.6%	63.6%/36.4%	0.14
Race (C/AA/A/O)	36.4%/9.1%/36.4%/18.2%	45.5%/0%/18.2%/36.4%	0.40
IOP (mmHg)	12.0 ± 1.9	18.6 ± 2.9	< 0.001
CCT (µm)	518.5 ± 34.7	513.4 ± 29.9	0.69
MD (dB)	−6.66 ± 4.5	−4.40 ± 5.3	0.21
VFI (%)	86.7 ± 10.7	87.5 ± 18.2	0.88

LTDH, low-tension optic disc hemorrhages; HTDH, high-tension optic disc hemorrhages; F, female; M, male; C, Caucasian; AA, African American; A, Asian; O, others; IOP, intraocular pressure; CCT, central corneal thickness; MD, visual field mean deviation; VFI, visual field index. *Data are given as mean ± standard deviation or percentage.

**Figure 1:**
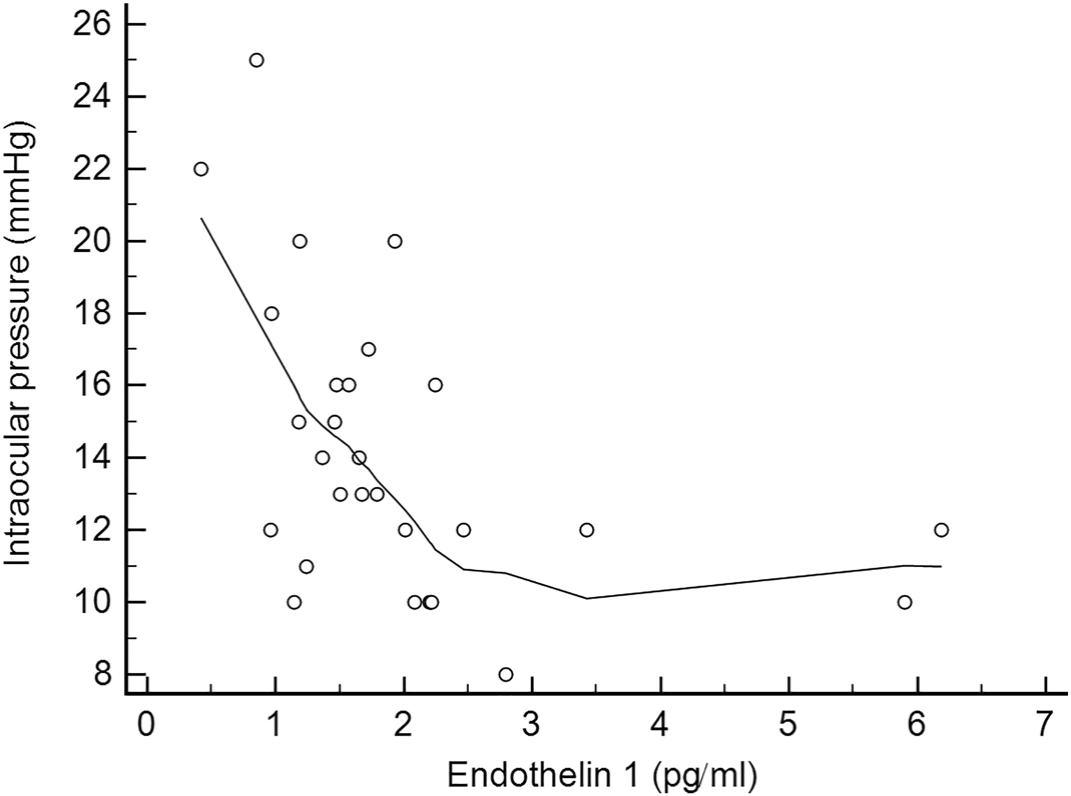
Scatter plot depicting a significant nonlinear correlation between endothelin-1 blood concentration and intraocular pressure at the time of disc hemorrhage detection. The plot was constructed using local regression smoothing trendlines (a span of 80% was adopted).

The ET-1 blood level was 65% higher in patients with LTDH compared to those with HTDH (2.27 ± 1.46 vs 1.37 ± 0.57 pg/ml, respectively; p = 0.03). In addition, a significant nonlinear negative correlation was found between ET-1 blood concentration values and IOP values at the time of DH detection (patients in the LTDH group presented higher ET-1 levels; r = −0.45, p = 0.02; Fig. 1).

**Figure 2:**
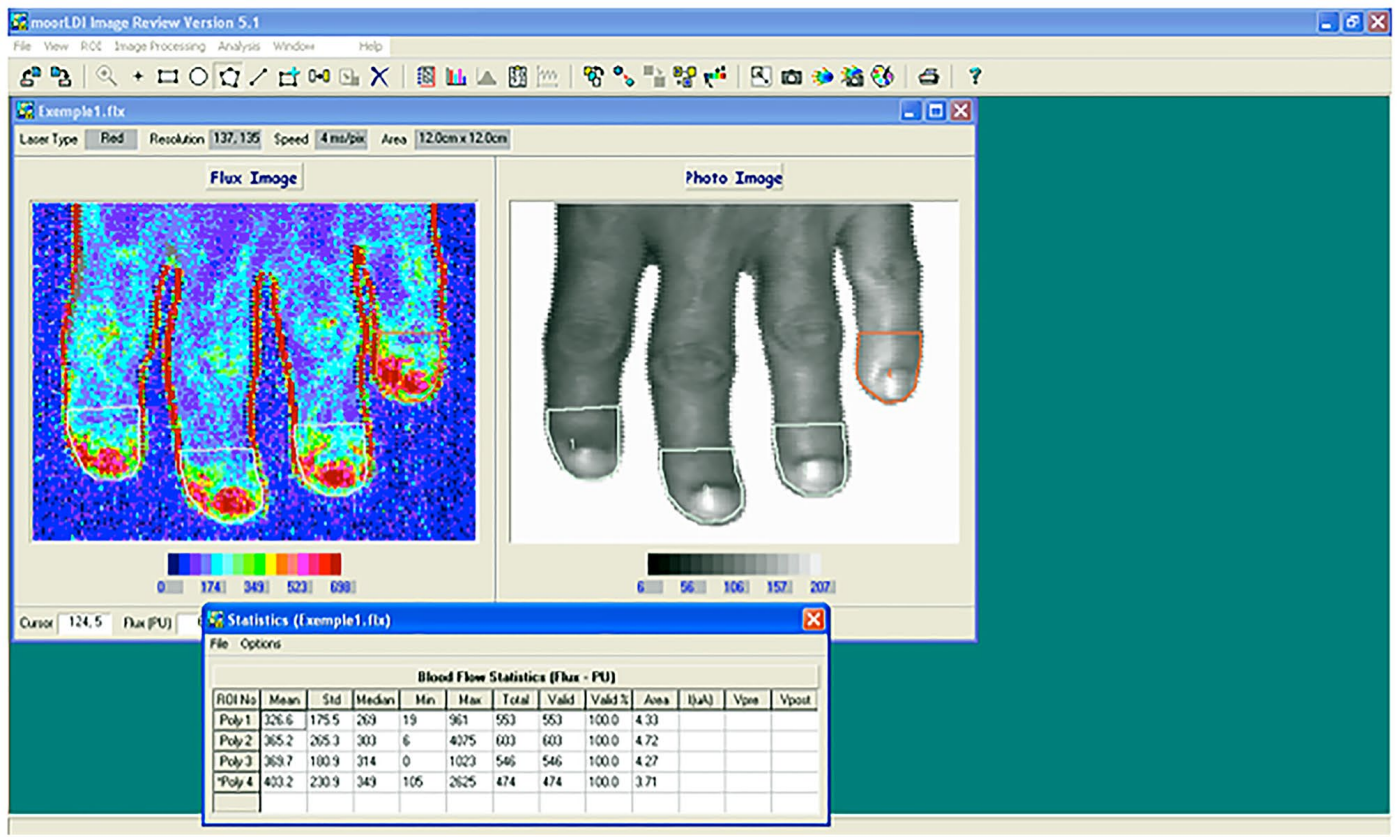
Laser Doppler imaging analysis of the distal phalanx (in this case of a patient presenting optic disc hemorrhage with an intraocular pressure of 18 mmHg).

**Figure 3:**
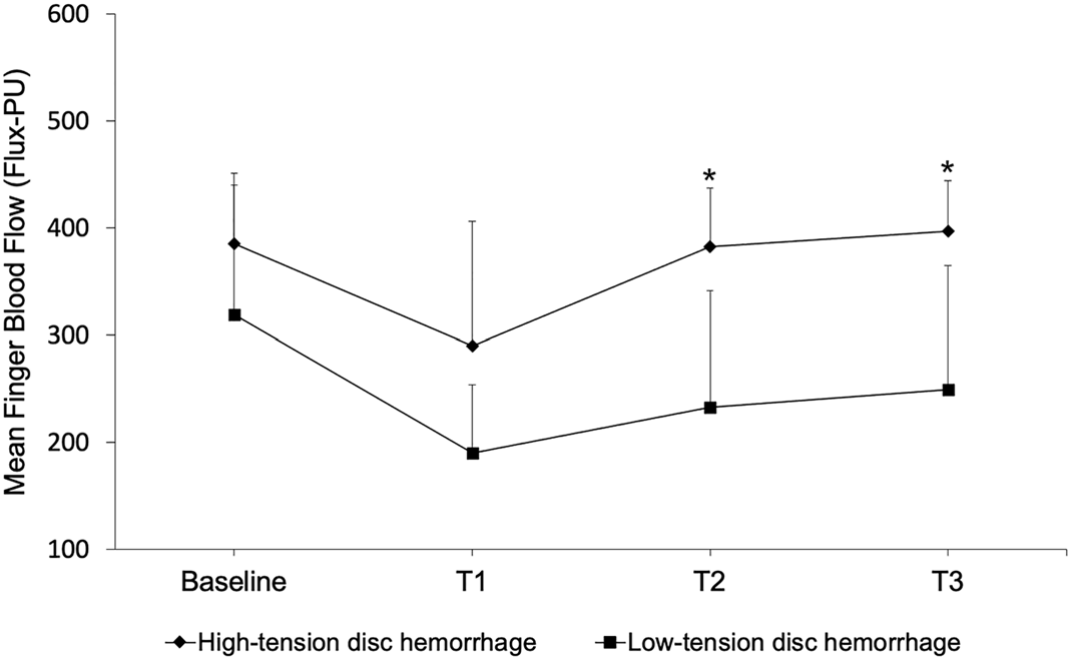
Mean finger blood flow estimated through laser Doppler imaging at baseline and the cold stimulus (T1, 1 min; T2, 10 min; T3, 20 min).

**Figure 4:**
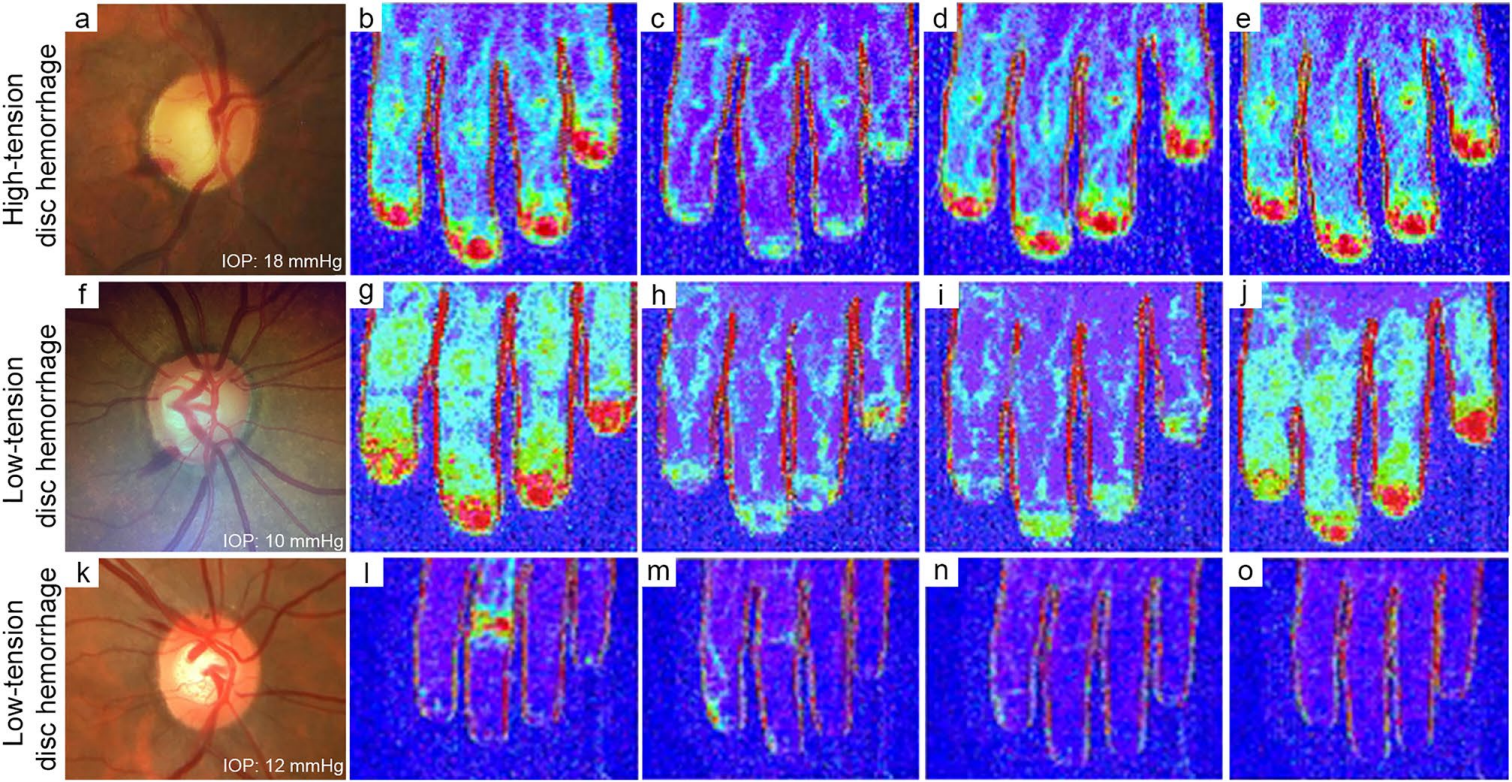
Example of three patients with disc hemorrhage. Disc photographs (**a**, **f**, **k**) and laser Doppler images before (**b**, **g**, **l**) and 1 (**c**, **h**, **m**), 10 (**d**, **i**, **n**), and 20 (**e**, **j**, **o**) minutes after the cold stimulus of patients with high-tension disc hemorrhage (**a**–**e**) and low-tension disc hemorrhage (**f–j** and **k**–**o**).

Blood flow measurements were estimated based on LD images (Fig. 2). In patients with LTDH, blood flow measurements were lower than those observed in patients with HTDH 10 min after the cold stimulus (233 ± 109 vs 382 ± 55 PU, respectively, p < 0.01) and persisted to be lower at 20 min (249 ± 116 vs 397 ± 47 PU, respectively, p < 0.01; Table 2 and Fig. 3). Figure 4 shows three examples of different LDI findings. Baseline and post-cold stimulation results at several time points are provided for patients with HTDH (a-e) or LTDH (f-o). For instance, note the slower recovery time documented in the patient in panels f-o (a case of LTDH) compared to that in panels a-e (a case of HTDH).

**Table 2:** Mean fingertip blood flow measured using laser Doppler imaging and capillaroscopy analysis in patients with low- and high-tension optic disc hemorrhages.

Variables*	LTDH	HTDH	p-value
Hemorrhage (Y/N)	14 (63.3%)/8 (36.4%)	6 (54.5%)/5 (45.5%)	0.61
Ectasia (Y/N)	15 (68.2%)/7 (31.8%)	6 (54.5%)/5 (45.5%)	0.44
Baseline FBF	319 ± 121	386 ± 66	0.15
T1 FBF	190 ± 64	290 ± 117	0.09
T2 FBF	233 ± 109	382 ± 55	0.001
T3 FBF	249 ± 116	397 ± 47	0.002
ΔΤ1 FBF (%)	−25 ± 22	−23 ± 28	0.87
ΔΤ2 FBF (%)	−25 ± 23	−0 ± 10	0.007
ΔΤ3 FBF (%)	−18 ± 18	−23 ± 13	0.49

LTDH, low-tension optic disc hemorrhages; HTDH, high-tension optic disc hemorrhages; Y, with hemorrhage/ectasia; N, without hemorrhage/ectasia; NC, nailfold capillaroscopy; FBF, mean fingertip blood flow; T1/T2/T3, 1/10/20 min after the cold stimulus, respectively; Δ, percentage difference between post- and pre-cold stimulus. *Data are given as mean ± standard deviation or number (percentage).

**Figure 5:**
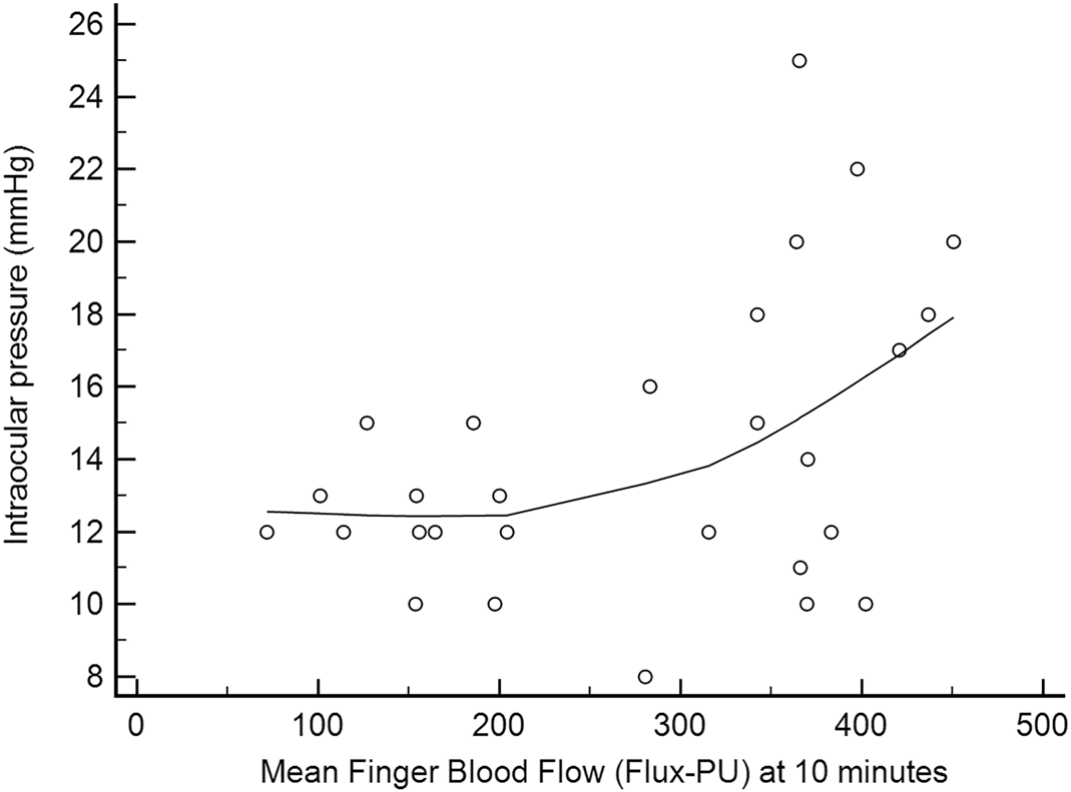
Correlation between blood flow measurement 10 min after the cold stimulus and intraocular pressure at the time of disc hemorrhage detection. The scatter plot was constructed using local regression smoothing trendlines (a span of 80% was adopted).

**Figure 6:**
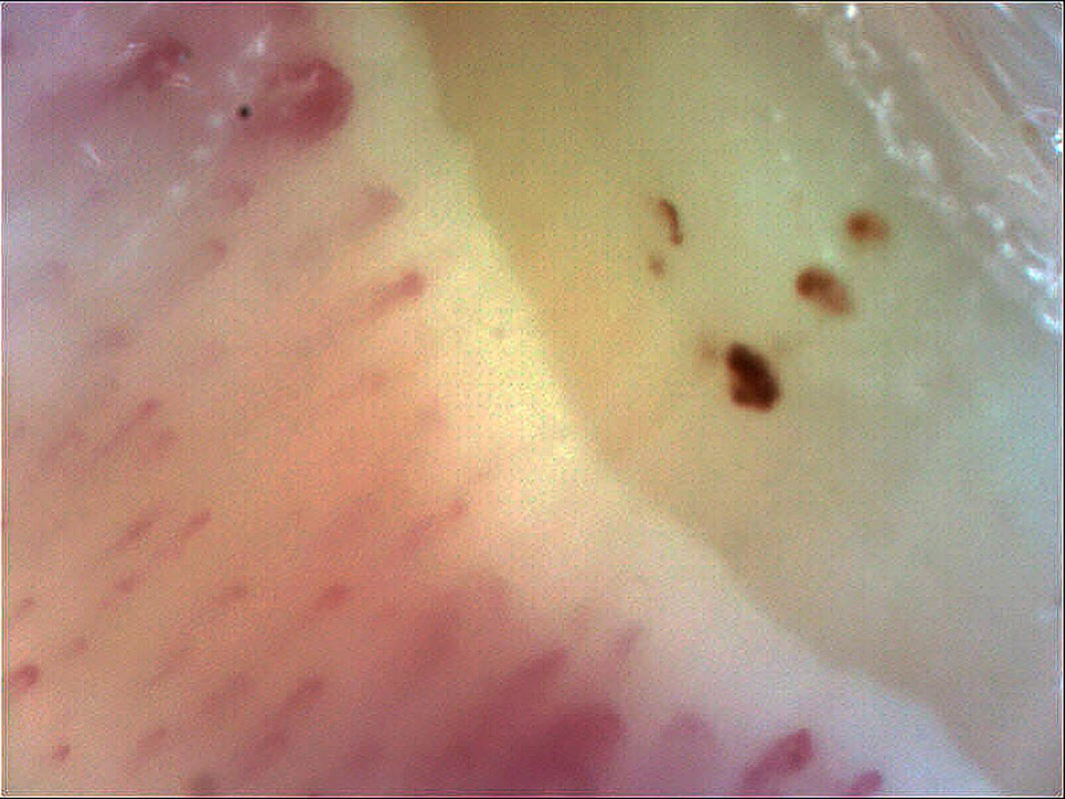
Nailfold capillaroscopy of a patient with low-tension disc hemorrhage showing three periungual hemorrhages.

In addition, a significant correlation between blood flow measurements 10 min after the cold stimulus and IOP values at the time of DH detection was found (patients in the LTDH group presented lower blood flow values; r = 0.45, p = 0.01; Fig. 5). Qualitative NC analyses showed the presence of hemorrhages (Fig. 6) and ectasia without differences between the LTDH and HTDH groups (P ≥ 0.44; Table 2).

The analysis between ET-1 blood levels and LDI values at 10 min after the cold stimulus showed a nominal negative correlation (r = −0.39, p = 0.07). Regarding vascular parameters and VF indexes, a negative correlation between ET-1 and visual field mean deviation (MD) was found (r = −0.44, p = 0.01). By contrast, ET-1 levels were not correlated with the visual field index (VFI; r = −0.30, p = 0.12). Likewise, LDI values at 10 min were correlated neither with MD (r = 0.06, p = 0.172) nor with VFI (r = −0.24, p = 0.24).

## Discussion

Both elevated IOP^4^ and DH occurrence^33^ have been consistently associated with glaucoma development and progression^11–13^. For example, Medeiros et al. evaluated rates of visual field progression in eyes with and without optic disc hemorrhages. Eyes with disc hemorrhages were found to have significantly faster rates of progressive visual field loss than those without disc hemorrhages, further supporting the concept of disc hemorrhages as an important risk marker for progression^34^. However, not every eye with a DH has a substantially elevated IOP. In daily clinical practice, many eyes with DH present with a seemingly well-controlled IOP^11–14^. This apparently contradictory scenario can be explained by the existence of different underlying DH mechanisms, such as IOP-induced (mechanical) or vascular-related glaucoma^15^. In the present study, evaluating different vascular metrics between patients with LTDH and those with HTDH, we found a greater prevalence of vascular abnormalities in those developing DHs with IOP values in the low teens. Not only had these patients a higher ET-1 blood concentration but also reduced blood flow measurements following cold stimulation. To the best of our knowledge, this is the first study to report on such findings in these specific groups of patients with DH.

Even though the concept of low- and high-tension DH was relatively recently introduced in the literature^35^, the investigation of vascular parameters in patients with glaucoma has substantially increased over the last years. Overall, our results corroborate in part the findings of previous studies that have pointed out vascular alterations (accessed by different methods) and ET-1 elevation in patients with glaucoma, especially in those with normal-tension glaucoma^19,36^.

Regarding the main findings of our study (ET-1, LDI, and NC results), we will separately address and contextualize each of our main findings. Concerning ET-1 levels, Grieshaber and Flammer previously associated serum ET-1 levels with the pathogenesis of DH in eyes with low IOPs, describing a more systemic context of primary vascular dysregulation^16^. Interestingly, in our study, not only did ET-1 levels differ between the study groups (patients with LTDH presented higher ET-1 levels than those with HTDH), but we also found a significant negative correlation with IOP values at the time of DH detection.

With regard to LDI, we did not find another study in which this method was used to evaluate glaucoma patients. The reproducibility of the LDI method was evaluated in a previous study of our group, showing an intraclass correlation coefficient of 0.980 (p < 0.001)^26^. Our findings suggest that compared to patients with HTDH, patients with LTDH are characterized by lower peripheral blood flow not only before cold stimulation, but they also have significantly and persistently lower values even 20 min after the cold stimulus, showing poor recovery. Although it would be reasonable to expect an association between ET-1 levels and LDI values from a clinical point of view and our study found a negative correlation between these parameters, we did not confirm a statistically significant correlation. This might be due to our study sample size which might not be powered enough to investigate this specific correlation.

In terms of NC analysis, we observed a high prevalence of vascular abnormalities in both groups, without differences between patients with LTDH and those with HTDH. This lack of difference corroborates the findings of another study that reported no differences in capillaroscopy findings comparing glaucomatous patients with and without DH history^37^. This observation can be explained by the fact that the presence of glaucoma itself (regardless of the glaucoma type) is already related to NC abnormalities, as reported by several studies^22,38–40^.

At this point, it is also important to discuss the main clinical implications of our findings. Our data suggest that glaucoma patients with low IOPs who develop DHs may present systemic vascular abnormalities. In the LDI, for instance, these patients not only had a lower initial response to the cold stimulus but also a poor recovery time. Moreover, patients presenting DH in the low teens had higher ET-1 levels than patients with high-tension DH, suggesting that patients presenting DH with apparently well-controlled IOP may present vascular dysregulation. On the other hand, patients with high-tension DH likely have a mechanical, pressure-induced DH mechanism. In addition, the negative correlation of ET-1 levels with IOP values at the time of DH detection suggests that in patients with higher ET-1 levels, high IOP values are not required for DH development. These findings suggest that distinct underlying mechanisms may be involved in patients developing DH with different IOP ranges and provide a basis for future studies focusing on individualized treatment modalities for each distinct mechanism.

The present study has some limitations that should be addressed. First, the baseline IOP was established based on a single measurement. Therefore, it is not known at which IOP the DH really occurred, only the presenting IOP when the DH was detected. As IOP varies considerably even in treated patients, an IOP profile based on multiple measurements for each patient is preferable. Second, our results are based on prospective data in a very specific population with strict well-defined inclusion and exclusion criteria. However, this led to a relatively small sample size, which limits the generalization and external validation of our findings. Third, due to the cross-sectional nature of our data, we can establish possible associations but not cause-effect relationships. Fourth, information on anticoagulant therapy was not collected. However, this parameter can influence DH occurrence. Finally, even though we have adopted a predefined and previously published definition of a glaucomatous DH in our study^41^, some patients with DHs related to other (non-glaucomatous) conditions might have been included. These facts should be considered when interpreting our findings.

## Conclusion

Patients developing DH with lower IOPs tend to have higher ET-1 blood levels and more peripheral vascular dysfunction as estimated by LDI compared to those with higher IOPs. These findings suggest that distinct underlying mechanisms may be involved in patients developing DH within different IOP ranges. We believe that our findings provide a basis for further investigations in larger and different populations which may confirm these initial results, bringing more information to help our understanding of these different underlying DH mechanisms, and hopefully shedding some light on this challenging topic.

## Data Availability

The datasets generated and analyzed during the current study are available from the corresponding author on reasonable request.

## References

[1] Kingman S (2004). Glaucoma is second leading cause of blindness globally. Bull. World Health Organ..

[2] Weinreb RN, Aung T, Medeiros FA (2014). The pathophysiology and treatment of glaucoma: A review. JAMA.

[3] Hollands H (2013). Do findings on routine examination identify patients at risk for primary open-angle glaucoma? The rational clinical examination systematic review. JAMA.

[4] Heijl A (2002). Reduction of intraocular pressure and glaucoma progression: Results from the Early Manifest Glaucoma Trial. Arch. Ophthalmol..

[5] Gordon MO (2002). The Ocular Hypertension Treatment Study: Baseline factors that predict the onset of primary open-angle glaucoma. Arch. Ophthalmol..

[6] Weinreb RN, Khaw PT (2004). Primary open-angle glaucoma. Lancet.

[7] Boland MV, Quigley HA (2007). Risk factors and open-angle glaucoma: Classification and application. J. Glaucoma.

[8] Yanagi M (2011). Vascular risk factors in glaucoma: A review. Clin. Exp. Ophthalmol..

[9] Grieshaber MC, Mozaffarieh M, Flammer J (2007). What is the link between vascular dysregulation and glaucoma?. Surv. Ophthalmol..

[10] Galassi F, Giambene B, Varriale R (2011). Systemic vascular dysregulation and retrobulbar hemodynamics in normal-tension glaucoma. Invest. Ophthalmol. Vis. Sci..

[11] Budenz DL (2006). Detection and prognostic significance of optic disc hemorrhages during the Ocular Hypertension Treatment Study. Ophthalmology.

[12] Bengtsson B, Leske MC, Yang Z, Heijl A, EMGT Group (2008). Disc hemorrhages and treatment in the early manifest glaucoma trial. Ophthalmology.

[13] Drance S, Anderson DR, Schulzer M, Collaborative Normal-Tension Glaucoma Study Group (2001). Risk factors for progression of visual field abnormalities in normal-tension glaucoma. Am. J. Ophthalmol..

[14] Jonas JB, Xu L (1994). Optic disk hemorrhages in glaucoma. Am. J. Ophthalmol..

[15] Kim KE, Park KH (2017). Optic disc hemorrhage in glaucoma: Pathophysiology and prognostic significance. Curr. Opin. Ophthalmol..

[16] Grieshaber MC, Flammer J (2007). Does the blood-brain barrier play a role in glaucoma?. Surv. Ophthalmol..

[17] Grieshaber MC, Flammer J (2005). Blood flow in glaucoma. Curr. Opin. Ophthalmol..

[18] Mozaffarieh M, Grieshaber MC, Flammer J (2008). Oxygen and blood flow: Players in the pathogenesis of glaucoma. Mol. Vis..

[19] Li S, Zhang A, Cao W, Sun X (2016). Elevated plasma endothelin-1 levels in normal tension glaucoma and primary open-angle glaucoma: A meta-analysis. J. Ophthalmol..

[20] López-Riquelme N (2015). Endothelin-1 levels and biomarkers of oxidative stress in glaucoma patients. Int. Ophthalmol..

[21] Lee NY, Park HY, Park CK, Ahn MD (2012). Analysis of systemic endothelin-1, matrix metalloproteinase-9, macrophage chemoattractant protein-1, and high-sensitivity C-reactive protein in normal-tension glaucoma. Curr. Eye Res..

[22] Kosior-Jarecka E (2018). Results of nailfold capillaroscopy in patients with normal-tension glaucoma. Curr. Eye Res..

[23] Lee NY, Park HY, Park SH, Park CK (2015). The association of nailfold capillaroscopy with systemic matrix metalloproteinase-9 concentration in normal-tension glaucoma. Curr. Eye Res..

[24] Park HY, Jung KI, Na KS, Park SH, Park CK (2012). Visual field characteristics in normal-tension glaucoma patients with autonomic dysfunction and abnormal peripheral microcirculation. Am. J. Ophthalmol..

[25] Murray AK, Herrick AL, King TA (2004). Laser Doppler imaging: A developing technique for application in the rheumatic diseases. Rheumatology (Oxford).

[26] Correa MJ, Andrade LE, Kayser C (2010). Comparison of laser Doppler imaging, fingertip lacticemy test, and nailfold capillaroscopy for assessment of digital microcirculation in systemic sclerosis. Arthritis Res. Ther..

[27] Camargo CZ, Sekiyama JY, Arismendi MI, Kayser C (2015). Microvascular abnormalities in patients with early systemic sclerosis: Less severe morphological changes than in patients with definite disease. Scand. J. Rheumatol..

[28] Almeida INF (2022). Clinical profiles of glaucomatous patients with high- and low-tension optic disc hemorrhages: A comparative study. J. Glaucoma.

[29] Leske MC (2007). Predictors of long-term progression in the early manifest glaucoma trial. Ophthalmology.

[30] Seissler J (2012). Vasoregulatory peptides pro-endothelin-1 and pro-adrenomedullin are associated with metabolic syndrome in the population-based KORA F4 study. Eur. J. Endocrinol..

[31] Corrêa MJ, Perazzio SF, Andrade LE, Kayser C (2010). Quantification of basal digital blood flow and after cold stimulus by laser Doppler imaging in patients with systemic sclerosis. Rev. Bras. Reumatol..

[32] Armitage P, Berry G, Matthews J (2002). Statistical methods in medical research.

[33] Shukla AG (2020). Disc hemorrhages are associated with the presence and progression of glaucomatous central visual field defects. J. Glaucoma.

[34] Medeiros FA (2010). The relationship between intraocular pressure reduction and rates of progressive visual field loss in eyes with optic disc hemorrhage. Ophthalmology.

[35] Lopes FSS, Junqueira DL, Biteli LG, Dorairaj S, Prata TS (2014). Clinical profiles of glaucomatous patients with high- and low-tension optic disc hemorrhages. Invest. Ophthalmol. Vis. Sci..

[36] Uz B (2016). Carotid arterial flow in pseudoexfoliation glaucoma and its role in diagnosing the disease. J. Glaucoma.

[37] Patel HY, Buys YM, Trope GE (2015). Nailfold capillaroscopy assessment in patients with glaucoma with a current optic disc hemorrhage. Can. J. Ophthalmol..

[38] Philip S, Najafi A, Tantraworasin A, Pasquale LR, Ritch R (2019). Nailfold capillaroscopy of resting peripheral blood flow in exfoliation glaucoma and primary open-angle glaucoma. JAMA Ophthalmol..

[39] Rong X (2020). Relationship between nailfold capillary morphology and retinal thickness and retinal vessel density in primary open-angle and angle-closure glaucoma. Acta Ophthalmol..

[40] Maric V (2019). Nailfold capillary morphology and platelet function in patients with exfoliative glaucoma. PLoS ONE.

[41] Prata TS (2010). Factors affecting rates of visual field progression in glaucoma patients with optic disc hemorrhage. Ophthalmology.

